# Advancements in daily precipitation forecasting: A deep dive into daily precipitation forecasting hybrid methods in the Tropical Climate of Thailand

**DOI:** 10.1016/j.mex.2024.102757

**Published:** 2024-05-31

**Authors:** Muhammad Waqas, Usa Wannasingha Humphries, Phyo Thandar Hlaing, Angkool Wangwongchai, Porntip Dechpichai

**Affiliations:** a The Joint Graduate School of Energy and Environment (JGSEE), King Mongkut's University of Technology Thonburi (KMUTT), Bangkok 10140, Thailand; b Center of Excellence on Energy Technology and Environment (CEE), Ministry of Higher Education, Science, Research and Innovation, Bangkok, Thailand; c Department of Mathematics, Faculty of Science, King Mongkut's University of Technology Thonburi (KMUTT), Bangkok 10140, Thailand

**Keywords:** Short term precipitation, Artificial intelligence, Neural networks, Deep learning, Forecasting, Wavelet transformation, LSTM, Advancements in Daily Precipitation Prediction

## Abstract

Climate change and increasing water demands underscore the importance of water resource management. Precise precipitation forecasting is critical to effective management. This study introduced a Daily Precipitation Forecasting Hybrid (DPFH) technique for central Thailand, which uses three different input-based models to improve prediction accuracy.

• The proposed methods precisely combine the biorthogonal wavelet transformation (BWT) function through BWT-RBFNN (Radial Basis Function Neural Networks) and (BWT-LSTM-RNN)Long Short-Term Memory Recurrent Neural Networks. Comparative analyses reveal that hybrid models perform better than conventional deep LSTM-RNN and Multilayer Perceptron Artificial Neural Networks (MLP-ANN). Although MLP-ANN showed moderate effectiveness, LSTM-RNN displayed notable enhancements, particularly evidenced by an impressive R^2^ (0.96) in Model M-2.
• The combination of BWT-LSTM-RNN yielded substantial enhancements, constantly surpassing standalone models. Specifically, DPFH-3 exhibited superior performance across multiple observation stations.
• The findings emphasize the efficiency of the BWT-LSTM-RNN models in capturing varied precipitation patterns, highlighting their potential to significantly improve the accuracy of precipitation forecasts, particularly in the context of water resource management in central Thailand.

## Graphical abstract

**Figure:**
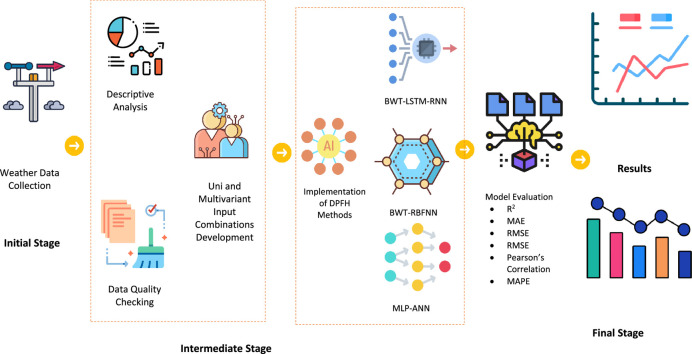

Specifications table

**Table:** 

Subject area:	Engineering
More specific subject area:	*Modeling and Forecasting*
Name of your method:	*Advancements in Daily Precipitation Prediction*
Name and reference of original method:	*NA.*
Resource availability:	*Data used to support the study's findings can be obtained from the corresponding author upon request.*

## Background

Precipitation forecasting signifies a crucial and complex component within the hydrological cycle, particularly during the current global climate change scenario [1]. The intricate spatiotemporal interrelations pose a critical challenge to achieving precise precipitation forecasts [2]. Accurate DPF (daily precipitation forecasting) is crucial for water resource planning, efficient irrigation management, runoff modeling, and crop protection applications [[2], [3], [4], [5]]. Previous investigations have explored several techniques to address the demands of DPF models. Two primary approaches are commonly employed: (a) data-driven techniques (DDTs), which utilize existing data to identify and classify patterns without presumptions, thereby adapting to present data patterns, and (b) model-driven techniques (MDTs), which involve establishing relationships between pertinent variables through structured models to generate predictions [6]. Model-driven techniques, such as numerical weather prediction (NWP), encompass approaches like simulating and forecasting atmospheric conditions through numerical techniques. These models mathematically represent the physical processes of the atmosphere, thereby enhancing our comprehension of weather patterns and phenomena [7,8]. While NWP models have demonstrated considerable success, they still display constraints in critical applications [9]. Their extensive computational requirements present a significant constraint, particularly in scenarios necessitating probabilistic forecasts, which often involve fewer than 50 ensemble members [10]. DDTs have noticed extensive application, particularly in DPF, to address hydrological challenges [[11], [12], [13], [14]]. Unfortunately, data-driven models (DDMs) possess limitations, including their empirical nature, which developed within a “black box” framework, and their susceptibility to overfitting [5,15]. Consequently, there has been a flourishing interest in leveraging artificial intelligence-based techniques (AITs) like machine learning (ML) to enhance and expedite NWP through DDTs [10]. AITs such as deep learning (DL), a subset of ML relying on complex artificial neural networks (ANNs), have demonstrated significant efficacy as a potent tool across a diverse array of tasks, with forecasting standing out prominently [5,16,17]. More recently, DL methods radial basis function neural network (RBFNN) [18], recurrent neural network (RNN) [[19], [20], [21]], and long short-term memory recurrent neural network (LSTM-RNN) [14,17,22] have also been used in precipitation forecasting. Globally, a predominant focus in existing research has been on applying these AITs for short-term (specifically daily) precipitation forecasting. For example, Endalie, Haile, and Taye (2022) introduced a deep LSTM model for daily rainfall prediction in southwestern Oromia, Ethiopia. Comparative analysis with existing models, including multilayer perceptron (MLP), k-nearest neighbors (KNN), support vector machine (SVM), and decision tree (DT), revealed the LSTM model's superior performance, achieving the lowest RMSE of 0.01 and higher R^2^, demonstrating its efficacy in daily rainfall prediction for Jimma [23]. A focus on monthly rainfall prediction in Simtokha, Bhutan, utilizing observation data, the predictive capabilities of various models, including Linear Regression, MLP, Convolutional Neural Network (CNN), LSTM, gated recurrent unit (GRU), and bidirectional LSTM. A novel BLSTM-GRU model surpasses existing ML and DL models, the proposed model outperforming conventional LSTM by 41.1 %, achieving a compared to LSTM's MSE (0.0128) [24]. The success accomplished by DL is accredited to the capacity of neural networks to recognize patterns within high-dimensional spaces. Improving the existing DL methods remains imperative, and one way to improve them involves the development of hybrid models. Integrating techniques like wavelet transformation (WT) with DL models such as LSTM RNN [25] and RBFNN is a viable approach to augmenting their performance [10,[26], [27], [28]]. The utilization of wavelet-based models has gained widespread acceptance for signal decomposition owing to the method's resilience and precision [29]. The approach's effectiveness is underscored by the robust multiresolution analysis characteristics inherent in the wavelet transform [30]. Only a few studies explored the DPF in Thailand [31,32].

Thailand, as a tropical country, is reliant on precipitation for its agriculture and economy [5]. Precipitation fluctuations, whether excessive or insufficient, can seriously affect the national economy and people's livelihoods. As a result, understanding the spatial and temporal patterns of precipitation is critical for a country's economic viability [33]. Approximately 13,100 acres of agriculture in Thailand are located outside irrigated zones, highlighting the importance of precipitation in the agricultural industry. This estimate shows that approximately 80 % of the nation's farming operations rely on precipitation [34,35]. Despite Thailand's agriculture's reliance on precipitation, there has been a noticeable lack of research in the region on advanced artificial intelligence algorithms for DPF. This gap emphasizes the need for novel techniques to overcome the issues of accurate precipitation forecasting in Thailand, especially given its economic importance and vulnerability to precipitation changes. Based on current literature, there is a huge gap where AITs can play a crucial role in DPF. Applying different AITs (wavelet transformation with ML, ANN, and DL techniques) can potentially increase the accuracy of DPF compared to those standalone AI models, which can improve this efficiency [5,25,36,37].

This study addresses the gap by providing hybrid Daily Precipitation Forecasting Hybrid (DPFH) algorithms that combine discrete wavelet transform (DWT) ensembles with deep LSTM-RNN and feedforward radial basis function neural networks (RBFNN). To develop effective and swift DPFH techniques and explore the potential improvements achievable in DPFH by leveraging ML methods and incorporating univariant and multivariant meteorological variables as inputs. The proposed techniques adopt hybrid DPFH techniques at 1-day, 2-day, and 3-day lagged datasets for combinations constructed based on data from 20 TMD observation stations. This research makes novel contributions in the following areas. First, novel DPFH models based on different discrete wavelet transform (DWT) family ensembles with deep LSTM-RNN and RBFNN are tested, which accounts for variability via time series univariant and multivariant decomposition. Second, the study confirms the suggested method's robustness by offering influential arguments backed up by the inclusion of several normalized measures for assessing and predicting accuracy. Third, the performance of the newly proposed methodology is rigorously compared to several forecasting methodologies, including hybrid and conventional deep LSTM-RNN and multilayer perceptron (MLP-ANNs) models. Three input variable combinations were built to develop an efficient model based on univariant and multivariant time series analysis.

## Method details

### Study Area and Dataset

**Fig. 1:**
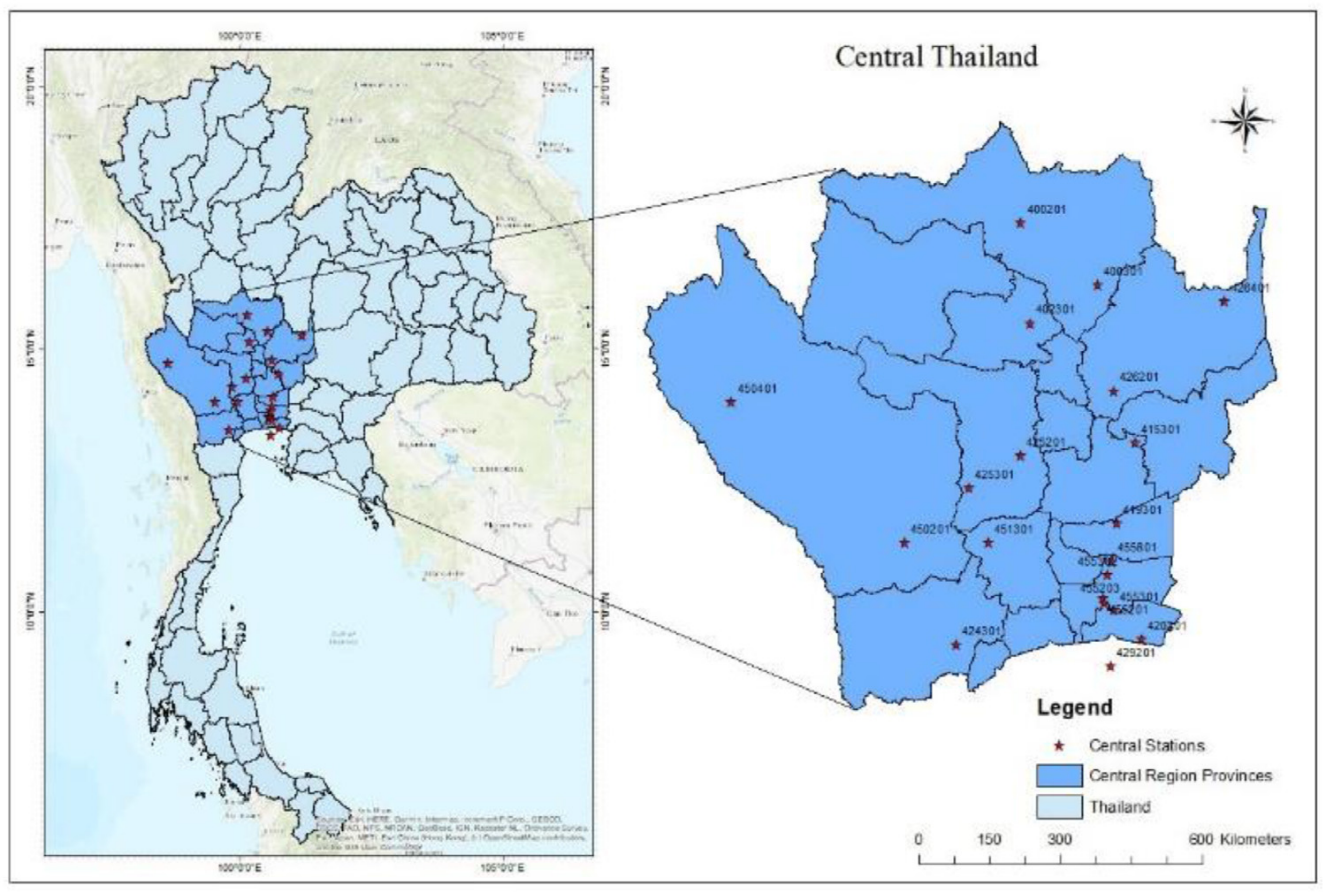
Distribution of observation stations over the central region of Thailand.

Thailand is geographically located between the latitudes of 5°37′ and 20°27′ north and longitudes of 97°22′ and 105°37′ east, characterized by a tropical climate [5]. The Thai Meteorological Department (TMD) has classified Thailand into five distinct climatic regions: (a) northern, (b) northeastern, (c) central, (d) eastern, and (e) southern regions. This study focused on Thailand's central region, which is recognized as a key agricultural and economic hub where livelihoods are inextricably linked to precipitation patterns. Fig. 1 shows the distribution of TMD stations over central regions of Thailand.

**Fig. 2:**
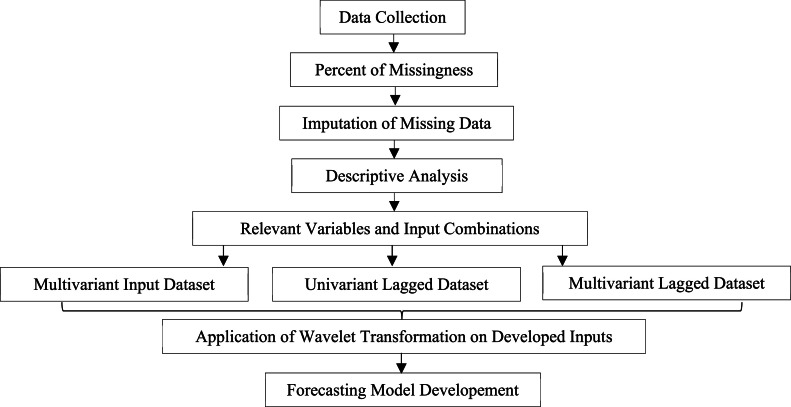
Flow diagram for data quality, and preprocessing to develop precipitation forecast model.

Daily precipitation (PPT), minimum temperature (T_min_), maximum temperature (T_max_), relative humidity (R_H_), and wind speed (W_S_) datasets from 20 TMD observation stations were gathered from 1993 to 2022. This research ensured data quality by checking missing values before conducting descriptive analyses as presented in Fig. 2. This study used the LSTM-RNN method, as Wangwongchai proposed (2023), to attribute any missing data [4]. The precipitation data can be damaged by repeated observational or processing errors within a hydrological year, necessitating stringent data consistency checks [38]. This study removed data from stations with over ten consecutive days of failure or exhibiting outlier values to ensure data integrity. However, when analyzing historical data, observation is crucial due to the potential impact of climate change, introducing irregular precipitation patterns deemed unusual by traditional statistical methods [39]. Employing the Grubbs and Beck (1972) technique, this study identifies critical values deviating significantly from the typical dataset range [40]. After applying this criterion to the initial dataset, descriptive daily precipitation statistics across 20 TMD stations are presented in Table 1.

**Table 1:** General information of the study area and descriptive statistics of daily precipitation over the region from 1993-2022.

Observation Station Name	Abbreviation	Station Code	Long	Lat	Province	Mean	SD	Min	Max	Skewness	C_V_
Nakhon Sawan	NK	400201	100.1355	15.66997222	Nakhon Sawan	2.43	8.73	0	133.90	5.93	358.86
Tak Pha Agro	TPA	400301	100.5305135	15.34971356	Nakhon Sawan	2.92	9.40	0	202.00	7.03	321.61
Chai Nat Agro	CAN	402301	100.1833333	15.15	Chai Nat	2.24	8.01	0	111.50	6.00	357.19
Ayutthaya Agro	TPA	415301	100.7277778	14.53333333	Ayutthaya	3.00	9.49	0	144.60	5.48	316.65
Pathumtani Agro	AG	419301	100.6333333	14.11666667	Pathum Thani	3.77	9.98	0	180.50	5.28	264.61
Ratchaburi Agro	RA	424301	99.7975	13.48722222	Ratchaburi	3.42	9.76	0	304.90	7.12	285.55
Suphanburi	SB	425201	100.1333333	14.46666667	Suphan Buri	2.97	9.29	0	190.40	5.85	313.09
U Thong Agro	UTA	425301	99.86666667	14.3	Suphan Buri	2.75	8.88	0	161.90	5.91	323.09
Lopburi	LB	426201	100.6166667	14.8	Lop Buri	3.10	10.06	0	164.90	5.70	325.05
Bau Chum	BC	426401	101.1908611	15.26494444	Lop Buri	3.32	10.10	0	173.10	5.74	304.14
Bangkok Pilot	BKKP	429201	100.5994444	13.37722222	Samut Prakan	2.86	9.09	0	153.30	5.79	318.31
Samut Prakan Agro	SPA	429301	100.6805556	13.44694445	Samut Prakan	1.88	7.62	0	124.00	7.24	404.27
Samut Prakan	SP	420201	100.7616667	13.51666667	Samut Prakan	1.34	5.10	0	117.70	9.34	380.76
Kanchana Buri	KB	450201	99.53333333	14.01666667	Kanchanaburi	2.50	8.20	0	132.40	6.50	327.80
Thongphaphum	TPP	450401	98.63638889	14.74222222	Kanchanaburi	4.57	9.64	0	142.50	4.52	211.20
Kampaeng Saen	KS	451301	99.96666667	14.01666667	Nakhon Pathum	2.13	8.08	0	146.40	6.67	379.06
Bangkok	BKK	455201	100.56	13.72638889	Bangkok	4.26	11.38	0	216.80	5.81	267.31
Klong Toey	KT	455203	100.5680556	13.70694444	Bangkok	3.77	10.95	0	242.60	6.44	290.87
Bang Na Agro	BNA	455301	100.61667	13.66667	Bangkok	4.12	10.84	0	185.90	5.08	262.93
Don Muang	DM	455601	100.605	13.919167	Bangkok	4.28	11.50	0	210.70	4.82	268.92

Both univariate and multivariate time series analyses are commonly employed in meteorological forecasting. The initial analysis was confined to solely utilizing historical daily precipitation values as input [34]. It can be represented as P(t-1), P(t-2), P(t-3), ..., P(t-n). This methodology examines historical precipitation data at different time lags to predict the precipitation value at the current time point, denoted as ``y.'' The P(t-1) signifies the precipitation value at the time point immediately preceding t, and this pattern extends up to P(t-n) [41]. This approach recognizes the temporal dependencies inherent in weather patterns, as past precipitation often influences current and future conditions [42]. The second analysis focused on important input multiple variables like Tmin, Tmax, RH, WS, and other available datasets. As mentioned by [2], these variables are critical in precipitation forecasting. Based on the literature [2,18,[43], [44], [45]], these input variables were selected, and three combinations were developed based on univariate and multivariant time series analysis mentioned in Table 2.

**Table 2:** Combinations of Model-based on Input variables.

Model-based on Input	Input Variables	Time Series Analysis
M-1/DPFH-1	PPT, Tmin, Tmax, RH, WS	Multivariant
M-2/DPFH-2	Pt, P(t-1), P(t-2), and P(t-3)	Univariant lagged dataset
M-3/DPFH-3	PPT, RH, Tmin, Tmax, WS, PPT_lag1, PPT_lag2, PPT_lag3, RH_lag1, RH_lag2, RH_lag3, Tmin _lag1, Tmin _lag2, Tmin _lag3, Tmax_lag1, Tmax_lag2, Tmax_lag3, WS_lag1, WS_lag2, and WS_lag3	Multivariant and Univariant lagged dataset

### Wavelet Transformation Functions (WTFs)

**Fig. 3:**
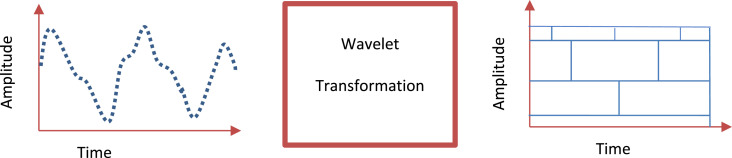
General Wavelet Transformation Process.

Many researchers applied mathematical transformations such as WTFs to time series datasets to extract additional information that may not be readily apparent from the raw data in its original time domain [[46], [47], [48], [49], [50], [51], [52], [53], [54]]. The WTF was introduced by Grossmann and Morlet in 1984. This method enables time series data analysis by providing time and frequency information, resulting in a time-frequency representation [46]. In the WTF context, the wavelet is a window function that exhibits oscillatory behavior and has a finite duration. It contrasts the sinusoids utilized in the Fourier transform (FT), which have an infinite duration [46]. The WT can analyze non-stationary data due to the varying scale of the WTF, which is computed for each spectral component. Fig. 3 demonstrates that varying the window size in WTF improves accuracy in time and frequency analyses, allowing for decomposing time series data into components of different resolutions. The mother wavelet, denoted as WT(t), exhibits a finite energy property, which can be formally expressed through mathematical notation as follows:

(1) $$\int_{-\infty }^{\infty }\psi \left(t\right)dt=0$$

The wavelet function, denoted as ψ_a,b_(t), is derived utilizing the following equation:

(2) $$\psi_{a,b\;}\left(t\right)={\left|a\right|}^{-\frac{1}{2}}\psi \left(\frac{t-b}{a}\right)$$

The variables ``a'' and “b” represent numerical parameters governing translation, scale, and frequency within wavelet analysis. The Wavelet Transform (WT) is mathematically formulated with a function dependent on both ``a'' and ``b.'' In this context, ``a'' determines the scale of the wavelet function ψ(t), contracting (a < 1) or dilating (a > 1) it, while ``b'' dictates temporal displacement. Temporal data analysis employs two methodologies: translation, shifting the wavelet along the temporal axis, and transformation, adjusting data via compression or expansion of the wavelet (also known as scale or dilation). Methods such as Continuous Wavelet Transform (CWT) overcome Short-Term Fourier Transform (STFT) limitations by employing variable window sizes for improved adaptability and efficacy. Conversely, Discrete Wavelet Transform (DWT) computes coefficients for dyadic scales and translations, enhancing data analysis efficiency. Table 3 comprehensively overviews various Wavelet Transform Functions (WTF) and their families.

**Table 3:** Types of Wavelet Transformation and Different Wavelet Families.

Continuous Wavelet Transform (CWT)	Discrete Wavelet Transform (DWT)	Wavelet Families	Sources
The CWT is an improved version of Short-Term Fourier Transformation (STFT) that addresses the STFT's defined window size issue [55]. **Mathematical Expression:** $\text{CW}T_{a,b\;}\left(t\right)=\int_{-\infty }^{+\infty }f\left(t\right)\frac{1}{\sqrt{a}}\psi *\;(\frac{t-b}{a}$) dt where “a” and “b” are dilation (scale) and translation (position) parameters, ψ(t) is the wavelet function, and f(t) is the original data. **Convolution:** Integration of the product of wavelet Ψ(t) and function f(t) across the temporal data range. **Scale Parameter (a):** Determines the scale of the wavelet, capturing signal details at different frequencies. A smaller scale is used for high-frequency components, and a larger scale is used for low-frequency components. **Visualization:** CWT coefficients (CWT_a,b_(t)) are represented as contour maps (scalograms), indicating similarity values ranging from 0 (no similarity) to 1 (complete similarity).	Converting a signal into a series of wavelets with discrete values is called the DWT, as defined by [51]. **Mathematical Expression:** $\text{DWT}\left(m,n\right)=2^{-\frac{m}{-2}}\sum_{t-0}^{N-1}\psi *\left(2^{-m}-2\right)\;x\;f\;\left(t\right)$ Where “m” and “n” are integers representing scale and translation, “a_o_” is the scale step, and “b_o_” is the location parameter. **Decomposition:** Signal f(t) is decomposed into approximation sub-signal T̅(t) (representing large-scale, low-frequency components) and detail sub-signals Wm(t) (representing low-scale, high-frequency components) at various levels of resolution.	**Haar Wavelet:** The Haar wavelet was initially introduced by [56]. A step-like behavior is suitable for sudden changes in time series data. This approach is deemed suitable for time series data that exhibit sudden changes. The Haar wavelet is deemed disadvantageous due to its non-differentiability, which is also associated with its discontinuous nature.	[55,51,56]
		**Daubechies Wavelet:** The nomenclature of the wavelets is attributed to Ingrid Daubechies, who is credited with developing compactly supported orthonormal wavelets [48]. Compact, orthonormal wavelets (dbN) with different vanishing moments (zero moments). The higher the N, the more accurately it captures polynomial trends. The members of the Daubechies wavelet family are db2, db3, db4, db5, db6, db7, db8, db9, and db10.	[48]
		**Coiflets Wavelet:** Developed by R. Coifman, characterized by vanishing moments. The Coiflets family includes coif1 to coif4, each with a specific number of vanishing moments, enhancing computational efficiency in wavelet transformation [49].	[49]
		**Symlets Wavelet:** Modification of the Daubechies family, least asymmetric, with members like Sym2 to Sym8. It has a similar structure to Daubechies but with minimal phase, making it suitable for specific signal processing applications.	[57]
		**Meyer Wavelet:** The Meyer wavelet, the second orthogonal wavelet, was developed by (Meyer 1985). The Meyer wavelet (t) and (T) scaling functions are defined in the frequency domain.	[58]
		**Biorthogonal Wavelets (BWT):** The Haar wavelet is a singular orthogonal wavelet with linear phase features, yet it is possible to build BWT with a linear phase [59]. BWT is characterized by a unique pair of scaling functions and corresponding scaling filters employed in analytical and synthetic procedures. Additionally, the biorthogonal framework mandates using a complementary set of wavelets and their associated wavelet filters for analysis and synthesis purposes [60]. It is possible to use a wavelet with more vanishing moments during the analysis phase to achieve a sparser representation. Simultaneously, using a smoother wavelet during the reconstruction phase is recommended, increasing the adaptability and usability of BWT in signal processing applications [61].	[[59], [60], [61]]

### Wavelet transformed long short-term memory recurrent neural network (BWT-LSTM-RNN)

The conventional LSTM-RNN, a specialized time-cyclic neural network, tackles the long-term correlation problem in generic RNNs. Initially introduced by Hochreiter and Schmidhuber [62], LSTM gained prominence in DL and garnered scholarly attention in subsequent research [58]. The LSTM approach was expressly built for learning long-term dependencies by designing functional sections of the memory cell state to fix and overcome the shortcomings of classic RNNs [17]. Fig. 5 shows the process of an LSTM-RNN, providing an intricate depiction of a memory cell. The functionality of the memory cell is analogous to that of a gated leaky neuron, notably featuring the unique attribute of self-connection in the subsequent phase. The input gate leverages data to revise the cell state (Ct), incorporating the tanh layer to generate an updated value that may be subsequently added. After this, Ct undergoes further updates.

**Fig. 4:**
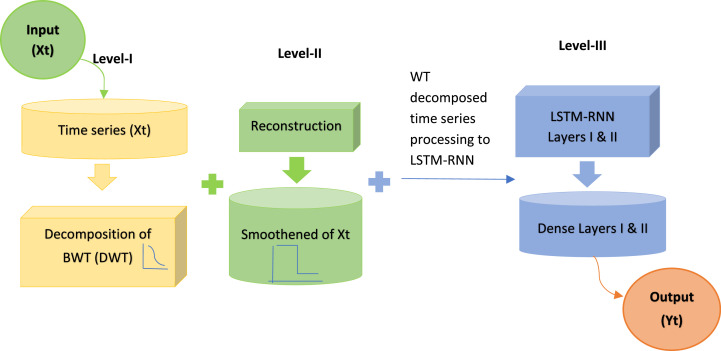
Hybrid BWT-LSTM-RNN working flow diagram.

**Fig. 5:**
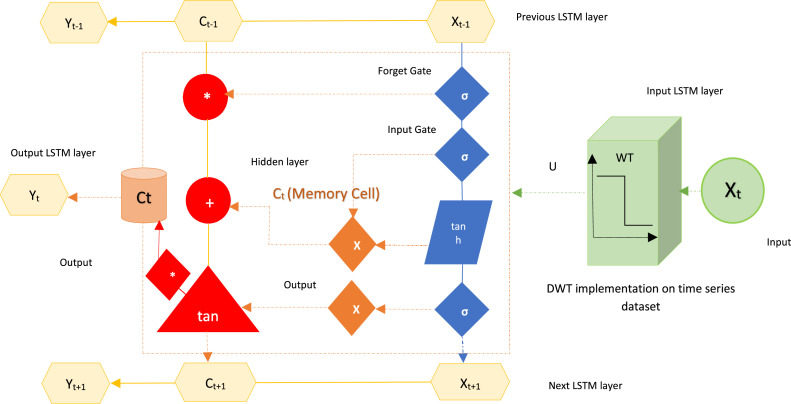
Architecture of Hybrid BWT-LSTM-RNN.

Moreover, the output gate regulates data transmission from Ct to the subsequent hidden layer, as elucidated by [3]. The BWT utilizing the bio1.3 family was selected after a comprehensive evaluation. This chosen methodology was combined and implemented alongside LSTM-RNN algorithms on different combinations of input variables (DPFH-1, DPFH-2, and DPFH-3) to achieve heightened accuracy in univariate and multivariate time series analyses. For LSTM-RNN, a deep model was intricately developed through hyperparameter search, identifying optimal configurations for each combination outlined in Table 2. The resulting Hybrid BWT-LSTM-RNN models encompass three levels, illustrated in Fig. 4. The first level involves BWT decomposition, where the bio1.3 wavelet decomposes the input univariate and multivariate time series. The second stage focuses on time series reconstruction, enhancing the predictive model's ability by reconstructing the input time series data. The third stage entails LSTM-RNN prediction, incorporating smoothed inputs, and deploying deep LSTM-RNNs. In the pursuit of optimal model calibration, the layers of wavelet decomposition must enhance the stability of detailed and approximate signals. Hence, in this research, the bio1.3 wavelet is employed, ensuring that the proposed hybrid BWT-LSTM-RNN model mitigates overfitting, ultimately surpassing the performance of the standalone LSTM model. Fig. 4, Fig. 5 schematically illustrate the WLSTM model, while Table 4 summarizes the hyperparameters of the best WLSTM model for predicting daily precipitation. In this analysis, 80 % of the dataset (1993-2016) was allocated for training purposes, while the remaining 20 % (2017-2022) was designated for testing the conventional and hybrid forecasting models. The governing equations for the hybrid BWT-LSTM-RNN are as follows:

(3) $$\begin{array}{ccc} Y_{t} & = & \left(X_{t}\;\cdot \;U_{o}+\;Y_{t+1\;}\cdot \;W_{o}\right)\;\cdot t\text{anh}\;\left(\sigma \left(X_{t}\;\cdot \;U_{f}+\;Y_{t-1\;}\cdot \;W_{f}\right)\right)\;\cdot \;C_{t-1}\\ & & +\;\sigma \left(X_{t}\;\cdot \;U_{i}+\;Y_{t-1\;}\cdot \;W_{i}\right)\;\cdot tanh\;\left(X_{t}\;\cdot \;U_{c}+\;Y_{t-1\;}\cdot \;W_{c}\right) \end{array}$$

**Table 4:** Exploration of hyperparameter search and identification of optimal model configurations.

Hyperparameters for Conventional LSTM-RNN		Optimal Model Configurations		
Parameters	Grid Search Range	M-1/DPFH-1	M-2/ DPFH-2	M-3/ DPFH-3
No. of hidden nodes	[10, 20, 30, 40, 50]	50	50	50
learning_rate	[0.1, 0.01, 0.001, 0.05, 0.005]	0.001	0.01	0.01
LSTM layer-1	[5, 10, 15, 20, 25,30]	20	20	20
LSTM layer-2	[5, 10, 15, 20, 25,30]	20	20	20
Dense layer-1	[10, 20, 30, 40,50]	50	50	50
Dense layer-2	[10, 20, 30, 40,50]	50	50	50
Output Layer	1	1	1	1
Activation function/layer	[‘tanh’, ‘relu’, ‘sigmoid’]	tanh, sigmoid	tanh, sigmoid	tanh, sigmoid
Optimizer	Adam	Adam	Adam	Adam
Verbose	[1, 2]	1	1	1
Batch Size	[12, 32, 64]	32	32	32
Epoch	[100, 300, 500, 800, 1000]	500	250	200
Loss	MSE	MSE	MSE	MSE

**Hyperparameters for Hybrid BWT-LSTM-RNN**				

Features	4, 3, 15	4	3	15
Responses	1	1	1	1
No. of hidden nodes	[10, 20, 30,40, 50]	50	50	50
Optimizer	Adam	Adam	Adam	Adam
learning_rate	[0.1, 0.01, 0.001, 0.05, 0.005]	0.01	0.005	0.005
Epoch	[100, 300, 500, 800, 1000]	500	1000	1000
Function to update the cell and hidden state	Tanh	Tanh	Tanh	Tanh
Function (gates).	Sigmoid	Sigmoid	Sigmoid	Sigmoid
Function (input weights)	Glorot (Xavier)	Glorot (Xavier)	Glorot (Xavier)	Glorot (Xavier)
Function (recurrent weights)	Bi-orthogonal	Bi-orthogonal	Bi-orthogonal	Bi-orthogonal
Level	[0,1,2]	1	1	1
Function to initialize the bias	Unit-forget-gate	Unit-forget-gate	Unit-forget-gate	Unit-forget-gate
LSTM layer-1	[10, 20, 30, 40,50]	50	50	50
LSTM layer-2	[10, 20, 30, 40,50]	50	50	50
Dense layer-1	[10, 20, 30, 40,50]	20	20	20
Dense layer-2	[10, 20, 30, 40,50]	20	20	20
Output Layer	1	1	1	1
Learning rate factor for the input, recurrent, and bias weights	**1**	**1**	**1**	**1**

**Hyperparameters for Hybrid BWT-RBFNN**				

No. of neurons	[50, 100, 150, 200]	100	100	100
Dense layer-1	[50, 100,200,300]	100	100	100
Dense layer-2	[50, 100,200,300]	100	100	100
Activation function	['constant,' 'adaptive,' logistic]	Logistic	Logistic	Logistic
RBF Sampler	Gamma [0.1,0.01,0.001, 10]	**0.001**	**0.1**	**10**
Random state	42	42	42	42
Scoring	MSE	MSE	MSE	MSE

**Hyperparameters for MLP-ANN**				

Hidden_layer_sizes	[(100,), (50, 50), (50,30,10)]	(50, 30, 10)	100	100
Activation functions	['relu', 'tanh']	relu	tanh	relu
Alpha	[0.0001, 0.001, 0.01]	0.0001	0.01	0.01
Learning_rate	['constant', 'adaptive']	constant	adaptive	adaptive
Max_iterations	[100, 500, 1000]	100	200	500
Random state	42	42	42	42
Scoring	Negative MSE	Negative MSE	Negative MSE	Negative MSE

### Wavelet transformed hybrid radial basis function neural network (BWT-RBF-ANN)

**Fig. 6:**
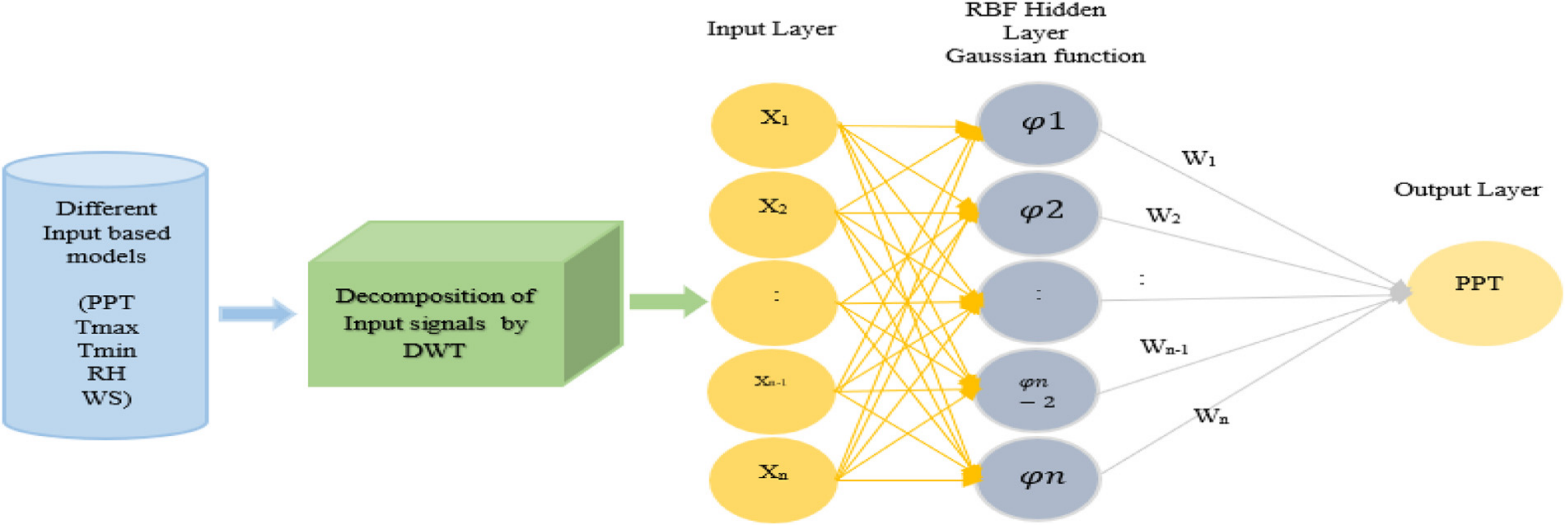
Working process diagram of Hybrid WT-RBF-ANN.

The RBF-ANN, introduced by Bromhead and Lowe in 1988 [63], is conceptualized based on the locally tailored responses observed in biological neurons. This model consists of three layers: input, hidden, and output, as illustrated in Fig. 6. The linkage between the input and output layers involves a nonlinear transformation, while the connection between the hidden and output layer spaces incorporates a linear transformation function [27]. The Gaussian function is a frequently employed choice for this purpose, particularly in its one-dimensional representation, as exemplified below:

(4) $$\varphi \left(x,\mu \right)=\;e^{\frac{∥x-\mu ∥{}^{2}}{2d^{2}}}$$

In the following expression, µ is the center of the Gaussian function, representing the mean value of x, and d is the distance (radius) from the center of φ(x, µ), providing a measure of the dispersion of the Gaussian curve. Significantly, the number of RBFs in the hidden layer is determined by the complexity of the mapping intended for modeling rather than the amount of the dataset. RBF-ANN uses a radial symmetric transfer function in the hidden layer, defined by radial symmetric transactions with centers and spread (r) parameters. The synaptic weights (wij) are determined only between the hidden and output layers [64]. The response of the jth node in the hidden layer (zj) to the input pattern Xj is expressed as follows:

(5) $$Z_{j\;}=\exp\left\{\frac{\left|X-\mu_{j}\right|}{\sigma_{j}^{2}}\right\}$$

The output of the network at the jth is determined as follows:

(6) $$Y_{L}=\sum_{j=1}^{L}Z_{j\;}*\;W_{ij}$$

The theoretical foundation of the RBF technique is based on the interpolation of multivariate functions, as defined by Cigizoglu in 2004. In the context of RBF, the solution to the exact interpolating mapping passes across each data point. This study provides more insights into RBF-ANN [65]. It has a unique architecture that provides various benefits, notably faster training than MLP-ANN. Another advantage is that the RBFNN has a two-stage training process, unlike the MLP-ANN, which uses supervised training approaches to determine parameters (biases and weights) simultaneously. This training process contributes to the RBFNN's efficiency and unique qualities [27].

### Multilayer perceptron artificial neural network (MLP-ANN)

**Fig. 7:**
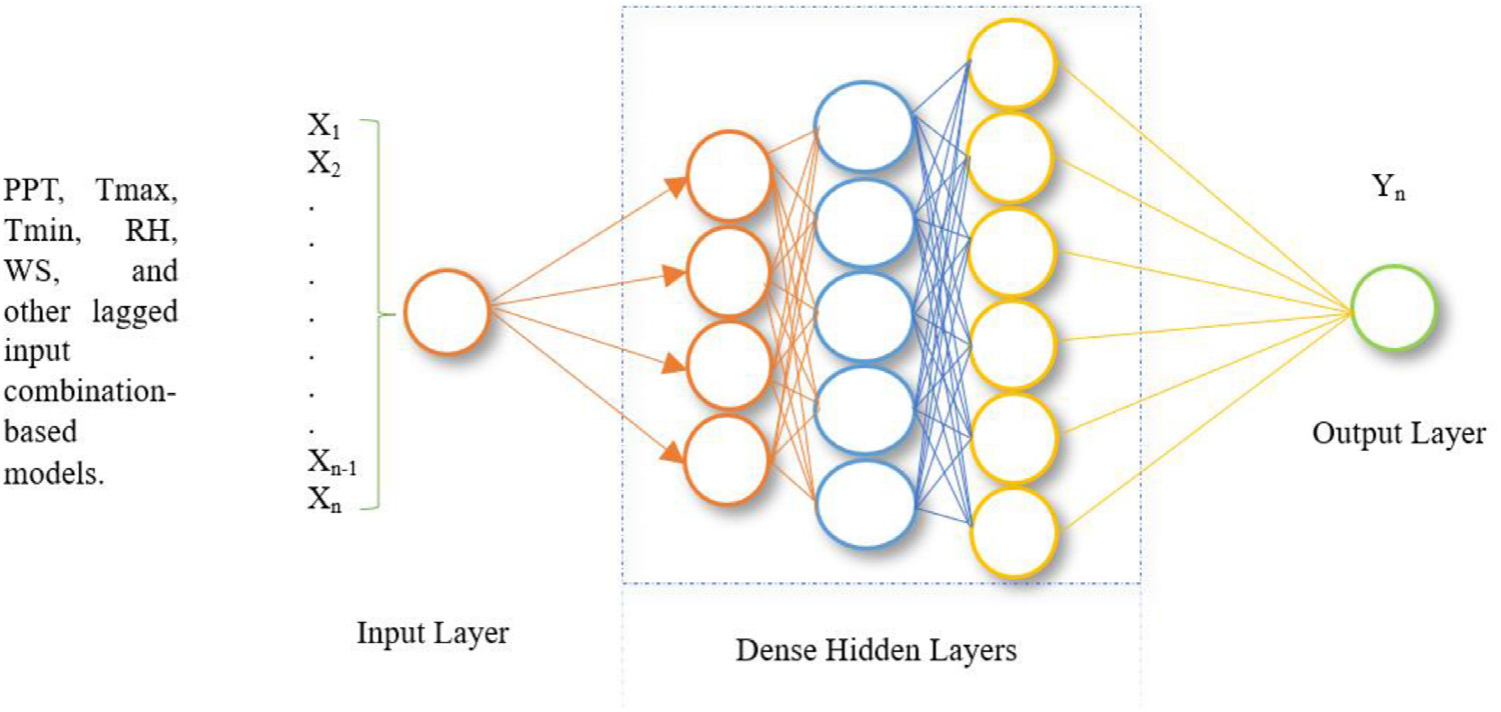
The architecture of Conventional Deep MLP-ANN.

The MLP-ANN is a conventional neural network [[66], [67], [68]]. The general architecture of an MLP-ANN is a feedforward structure with an input layer, one or more hidden layers, and an output layer, similar to RBF-ANNs, consisting of three layers [5]. The backpropagation algorithm is often used while training MLP-ANNs. Activation functions, such as linear, hyperbolic tangent (sigmoid), and logistic functions, are used to assess their impact on a multilayer perceptron. Each layer has a unique role in the overall operation of the network. Neurons in one layer communicate with neurons in the next layer via weighted connections, as shown in Fig. 7. In the illustration, x_1_, x_2_..., x_n-1_, and x_n_ indicate the ANN's inputs, whereas Y_n_ represents the desired output. Waqas et al. (2024) explain how the MLP-ANN works [2]. The deep MLP-ANN's hyperparameter search and optimal model configuration for this study are presented in Table 4.

### Evaluation metrics

To assess the capability of the model, this study uses statistical evaluation metrics such as Pearson's Correlation (r), mean absolute percentage error (MAPE), mean absolute error (MAE), root mean square error (RMSE), relative RMSE (RRMSE), mean absolute error (MAE), and coefficient of determination (R2). These five statistical parameters' formulae are as follows:

(7) $$R^{2}=1-\sum \frac{{\left(P_{obs}\;-\;P_{pre}\right)}^{2}}{{\left(P_{obs}-P_{avg}\right)}^{2}}$$

(8) $$\text{RMSE}=\frac{\sqrt{\sum_{i=1}^{N}{\left(\text{Pobs}-\text{Ppre}\right)}^{2}}}{N}$$

(9) $$\text{RRMSE}=\frac{\sqrt{\frac{1}{n}\sum_{i\;=\;1}^{n}{\left({\bar{P}}_{Obs}^{i}-{\bar{P}}_{pre}^{i}\right)}^{2}}}{\sum_{i=1}^{n}{\bar{PR}}_{Obs}^{i}}$$

(10) $$\text{MAE}=\sum \frac{\left(P_{pre}\;-\;P_{obs}\right)}{N}$$

(11) $${\text{Pearson}}^{\prime }s\;\text{correlation}\;\left(r\right)\;=\frac{n\left(\sum P_{obs}*P_{pre}\right)\;-\;\left(\sum P_{obs}\right)\left(\sum P_{pre}\right)}{\sqrt{\left[n\sum {P_{obs}}^{2}\;-\;\left(\sum {P_{pre}}^{2}\right)\right][n\sum {P_{pre}}^{2}-\left(\sum {P_{pre}}^{2}\right)}}$$

(12) $$MAPE=\;\frac{1}{n}\;\sum_{i=1}^{n}\left|P_{obs}-P_{pre}\right|*100$$

The coefficient of determination (R^2^) which varies between 0 and 1. An R^2^ approaching or reaching one indicates optimal model performance. Also, the Pearson correlation coefficient (r) spans from -1.00 to +1.00, with -1.00 representing a perfect negative correlation, +1.00 representing a perfect positive correlation, and 0.00 indicating no correlation between the variables [62]. The accuracy measure, termed relative RMSE, is derived by dividing RMSE by the mean of the measured data values. Model accuracy is classified as good when the relative RMSE falls within the range of 10 % to 20 %, deemed fair if it lies between 20 % and 30 %, and considered poor if the relative RMSE exceeds 30 %, as per the findings of Li, Tang et al. (2013) [2]. MAE, a measure of the average absolute errors divided by the number of observations, is crucial for assessing model performance [69]. The MAPE serves as a metric to assess the precision of predictive models, particularly in the context of forecasting and regression challenges. MAPE values generally span from 0 % (indicating a flawless forecast) to 100 % or higher (suggesting a substantial forecast error), with reduced values signifying enhanced forecast accuracy. Nominal RMSE values suggest a well-performing model [47]. MBE gauges average bias, indicating the need for bias correction, while MAE represents the difference between expected and observed values [2].

## Method validation

This section investigates innovative DPFH methodologies for short-term precipitation forecasting, integrating biorthogonal DWT ensembles with deep LSTM-RNN and RBFNN architectures. To train the model, the standalone and hybrid LSTM-RNN and RBFNN are trained on 80 % of the historical series, to adapt to precipitation patterns by trial and error. The best parameters chosen during calibration are then used for model validation, with simulations performed on the remaining 20 % of the historical data. The model's performance is evaluated by comparing observed data to simulations generated by both LSTM-RNN and RBFNN and utilizing error statistics (R^2^, RMSE, RRMSE, Pearson's Correlation, and MAPE). These comparative analyses showcase the proposed method's resilience and accuracy, highlighting its potential contributions to meteorological forecasting over conventional models like LSTM-RNN and MLP-ANN.

**Fig. 8:**
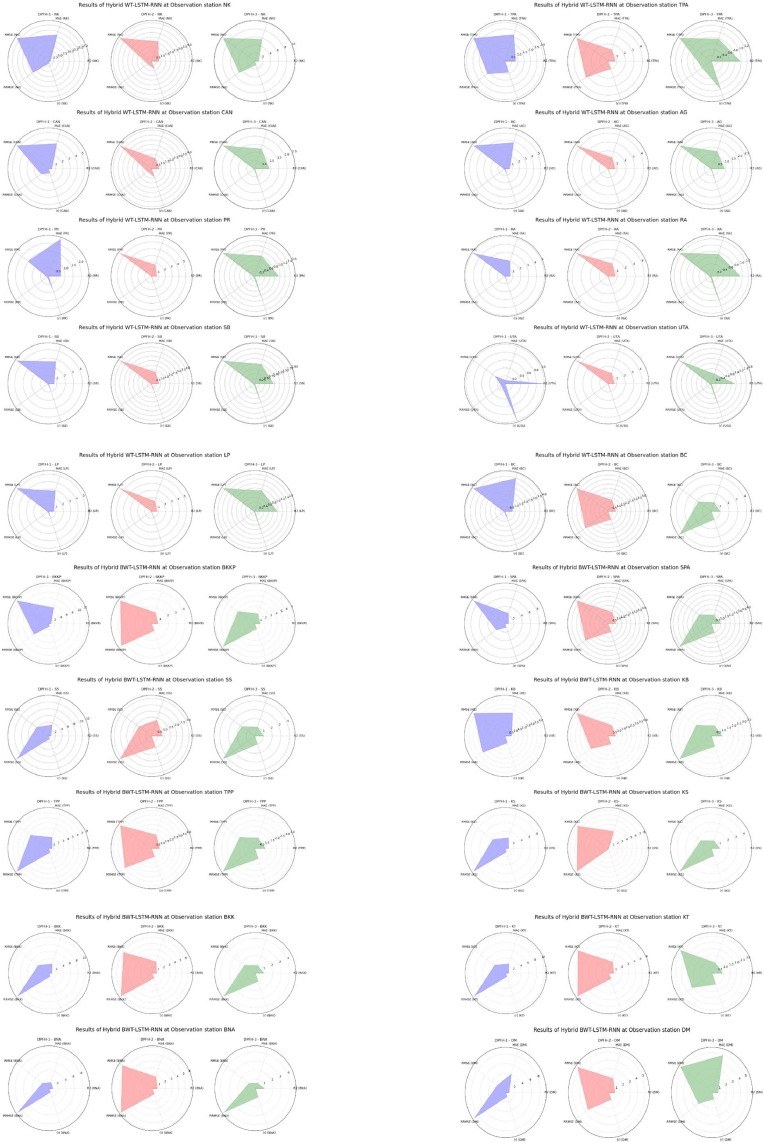
Comprehensive performance assessment of the developed prediction models (DPFH-1, DPFH -2, and DPFH -3) utilizing Hybrid WT-LSTM-RNN, conducted across TMD stations 1 to 20.

Fig. 8 shows the evaluation results of the hybrid BWT-LSTM-RNN precipitation forecasting models (DPFH-1, DPFH-2, and DPFH-3) over different observation stations. Using radar maps for visual interpretability provides an extra source of information on the spatial dynamics of each model's performance at specific observation stations [4]. Starting with the NK station, DPFH-3 is the best-established model in proficiency, with a high R^2^ of 0.98. The low values of MAE (5.7) and RMSE (9.66) restate the accuracy of prediction, implying minimal error exists in rainy-day forecasting using the statistical model adopted. The (r) 0.99 confirms the linear relationship between modeled and observed values. Continuing to assess and compare DPFH-3 performance at the TPA station, DPFH-3 still performs better with R^2^ (0.97), low MAE (0.78), RMSE (1.3), and high (r) of 0.99. For DPFH-1, the top performer will come out as the UTA station, which has an R^2^ of 0.99. The MAE and the RMSE are also ultra-minimal - the precipitation forecasts depict impeccable accuracy. Further, the model, with a (r) of 0.99, again shows the ability to support development by closely following observed values.

In contrast, at the KS station, where DPFH-2 has given inferior performance with the R^2^ (0.1), DPFH-3 rises to prominence, marked by the impressive value of 0.97. The low values of MAE and RMSE 0.93 and 1.53 indicate accurate precipitation forecasts, while a high (r) of 0.91. The overall radar map coverage quality at station NK appears promising, with some spatial discrepancy in line with its moderate R^2^ (0.92). This is further supported by the general precipitation patterns being captured well, albeit deviations from observed values can be seen when certain map parts are specified. The radar map of DPFH-2 shows a less consistent coverage, which calls for its relatively lower R^2^ (0.75). Lagged univariate data may find it challenging to catch temporal patterns perfectly well, hence fluctuations in rainfall prediction. DPFH-3 radar map features display a well-distributed and close-matching precipitation pattern, supporting its high R^2^ (0.98) and strong correlation (r = 0.99). Multivariate lagged datasets contribute to a comprehensive understanding of the precipitation's variations. Both stations, TPA, UTA, and KS, offer similar visual interpretations that measure how well each model captures the spatial and temporal precipitation patterns at these various locations. Expanding the analysis to additional stations such as CAN, AG, and PR, the radar maps show detailed coverage and accuracy for each model. The radar maps of DPFH-3 depict extensive and accurate radar precipitation coverage. The incorporation of multivariate datasets with lags in DPFH-3 appears salient for the prowess exhibited by the system in pinpointing complex associations, thereby notably enhancing the accuracy of relevant models.

**Fig. 9:**
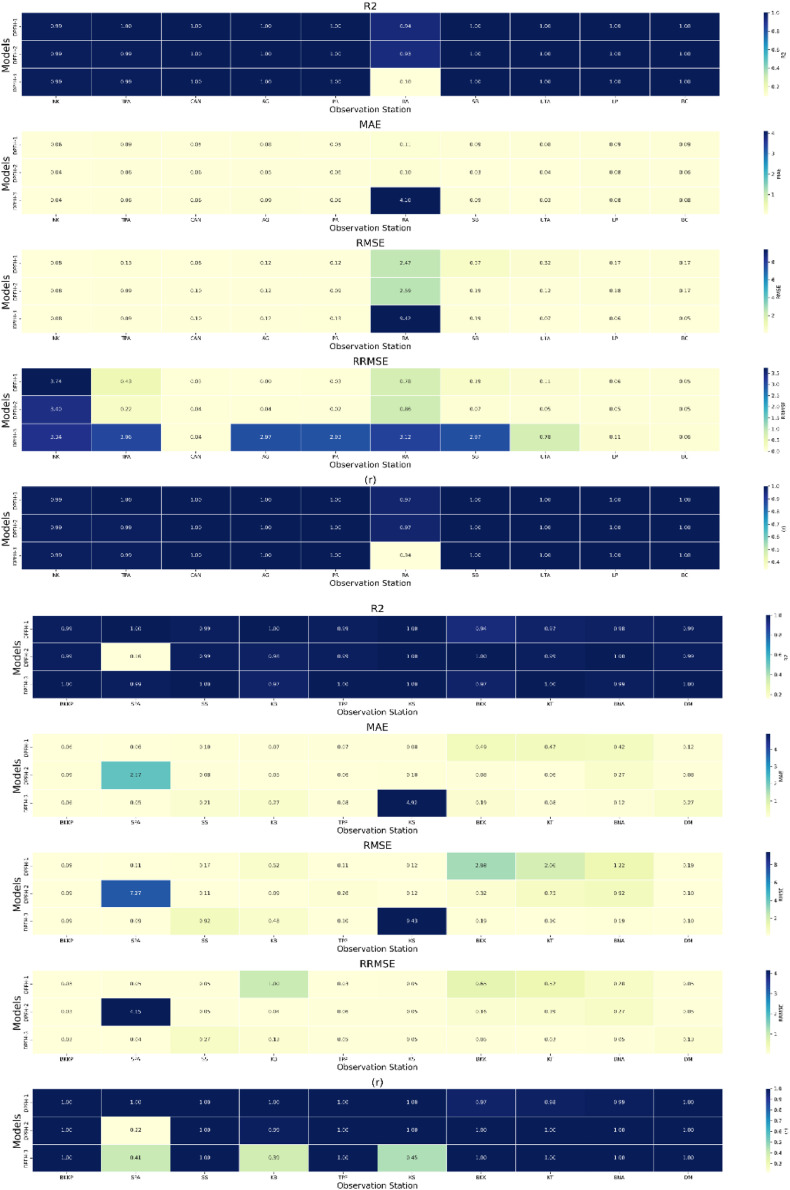
Comprehensive performance assessment of the developed prediction models (DPFH-1, DPFH -2, and DPFH -3) utilizing Hybrid WT-RBFNN, conducted across TMD stations 1 to 20.

Fig. 9 presents the evaluation of the precipitation forecasting hybrid BWT-RBFNN models. For the NK observation station, DPFH-1 and DPFH-2 show exceptional performance with R^2^ of 0.99, indicating a high level of explained variance. These models also exhibit low MAE (0.06 and 0.04, respectively) and RMSE (0.08), suggesting accurate predictions. However, DPFH-3 shows significantly lower performance, with an R^2^ of 0.17, high MAE (3.25), and RMSE (7.79), indicating poor model fit. At the TPA station, DPFH-1 and DPFH-2 again perform remarkably well with perfect R^2^ values of 1.00, indicating a perfect fit to the data.

Furthermore, the models exhibit low RMSE (0.13 and 0.09) and MAE (0.09 and 0.06), indicating high prediction accuracy. Conversely, DPFH-3 displays subpar performance, aligning with observations from the NK station. DPFH-1 and DPFH-2 consistently demonstrate exceptional efficiency, evidenced by an R^2^ of 1.00 and nearly zero MAE and RMSE values across stations such as CAN, AG, PR, SB, UTA, LP, and BC. Conversely, DPFH-3 exhibits an insignificant fit to precipitation data, consistently yielding higher MAE, RMSE, and R^2^ values. Notably, DPFH-2 exhibits outstanding performance at the BKKP station, with an R^2^ of 1.00, indicating an ideal alignment with observed data. Furthermore, it demonstrated low MAE (0.09) and RMSE (0.05) values, underscoring precise and reliable predictions. Strong performance was also indicated by high R2 (0.99) and low error metrics (RMSE = 0.06, MAE = 0.09) for DPFH-1. Conversely, DPFH-3 exhibited notably poorer performance, evidenced by an R^2^ of 0.10, a high RMSE of 3.88, and an RRMSE of 8.02, indicative of inadequate precision and reliability. Similar trends were observed across other sites. DPFH-2 consistently outperformed other models with an R^2^ of 1.00, showcasing robust predictive capabilities. In contrast, DPFH-3 consistently lagged, exhibiting more significant error metrics and lower R^2^ values than DPFH-1, demonstrating strong performance. Across various stations, DPFH-2 consistently delivered accurate and dependable precipitation forecasts according to the provided evaluation criteria. While DPFH-3 exhibited lower accuracy and higher error metrics, indicating limitations in capturing precipitation patterns, DPFH-1 demonstrated satisfactory performance. Comparative analysis with other multivariate and lagged datasets utilized in DPFH-1 and DPFH-3 suggests that the univariate lagged dataset (DPFH-2) is a reliable and effective approach for improving precipitation forecasting. The challenges associated with increasing complexity may explain the suboptimal performance of BWT-RBFNN when employed with multivariate DPFH-1 and DPFH-3. Multicollinearity and redundant information hindered the ability of multivariate DPFH-1 to discern significant features crucial for precipitation forecasting promptly. Similarly, in DPFH-3, if the lagged variables fail to introduce meaningful noise or contribute substantially, the model may encounter challenges in accurately representing the temporal intricacies they entail. In contrast, the success of univariate DPFH-2 suggests that simplifying the input space by focusing on lagged precipitation data alone facilitates more accurate and robust predictions in the context of the BWT-RBFNN.

**Fig. 10:**
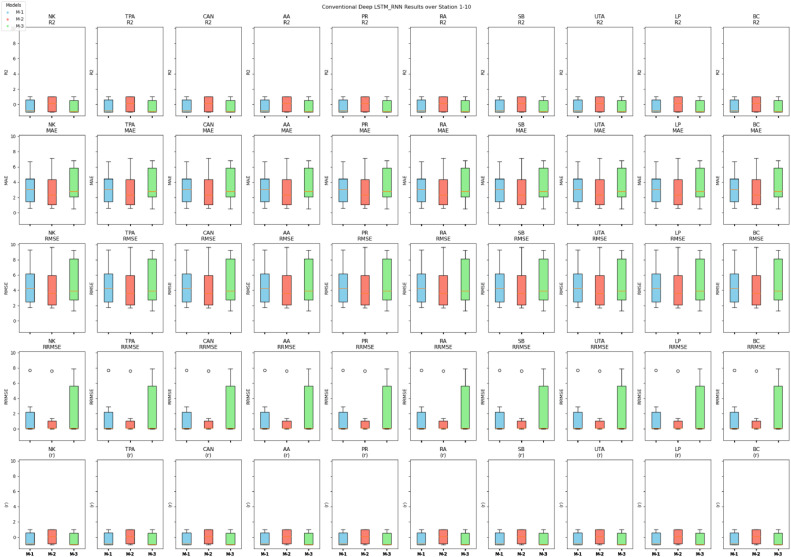
Comprehensive performance assessment of the developed prediction models (M-1, M-2, and M-3) utilizing conventional LSTM-RNN, conducted across TMD stations 1 to 10.

**Fig. 11:**
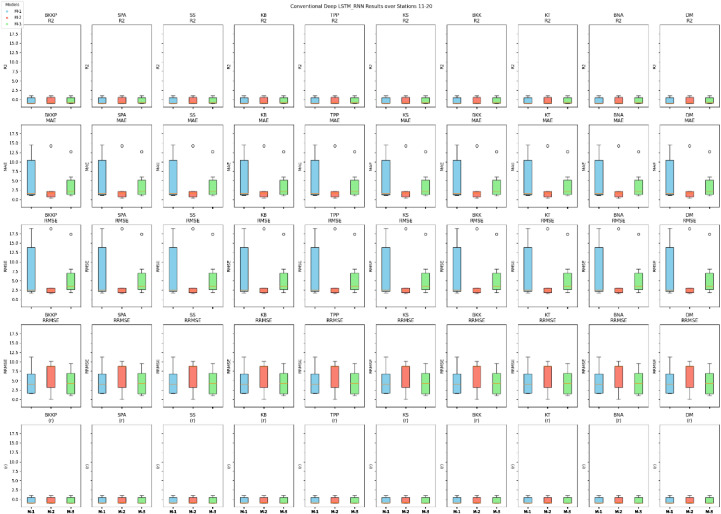
Comprehensive performance assessment of the developed prediction models (M-1, M-2, and M-3) utilizing conventional LSTM-RNN, conducted across TMD stations 11 to 20.

The evaluation outcomes of three models (M-1, M-2, and M-3) shown in Fig. 10, Fig. 11 across multiple observation stations, employing the conventional deep LSTM-RNN, yield significant insights. The R^2^ ranges from 0.50 to 0.98. Henceforth, Model M-2 exhibits notable performance with commendable R^2^ from 0.74 to 0.98, outperforming a significant portion of observation stations. This suggests the feasibility of capturing temporal dynamics within the precipitation process utilizing the univariate lagged dataset model, which incorporates precipitation's 1-day, 2-day, and 3-day time lag data. Furthermore, the efficacy of M-2 prediction accuracy is substantiated by low MAE values, ranging from 0.50 to 6.71, alongside RMSE, which varies from 0.01 to 9.64. On the other hand, Model M-1 exhibits mixed performance over the stations of observations, with R^2^ ranging from 0.55 to 0.98. Model M-3 also exhibits varied performance across the studies for R^2^ (0.50 to 0.97). MAE, RMSE, and RRMSE values are higher for M-1 and M-3 than for M-2. However, it is interesting to emphasize that the Model M-2 precipitation predicted series shows a high (r) varies from 0.87 to 0.99, meaning a strong linear relationship exists between forecasted and observed precipitation. Therefore, further enhances the reliability of M-2 in capturing the intricate precipitation patterns at different observation stations. In addition, R^2^, MAE, RMSE, and (r) in the comprehensive evaluation of the three-input combination-based models emphasize the superiority of forecasting accuracy of the univariate lagged dataset model (M-2) over the multivariate models (M-1 and M-3). MLP-ANN was inefficient in learning the relationship between multivariant variables at all stations and efficient with M-2, which consisted only of PPT and lagged data. Across all the metrics and stations, M-2 showed excellent performance.

**Fig. 12:**
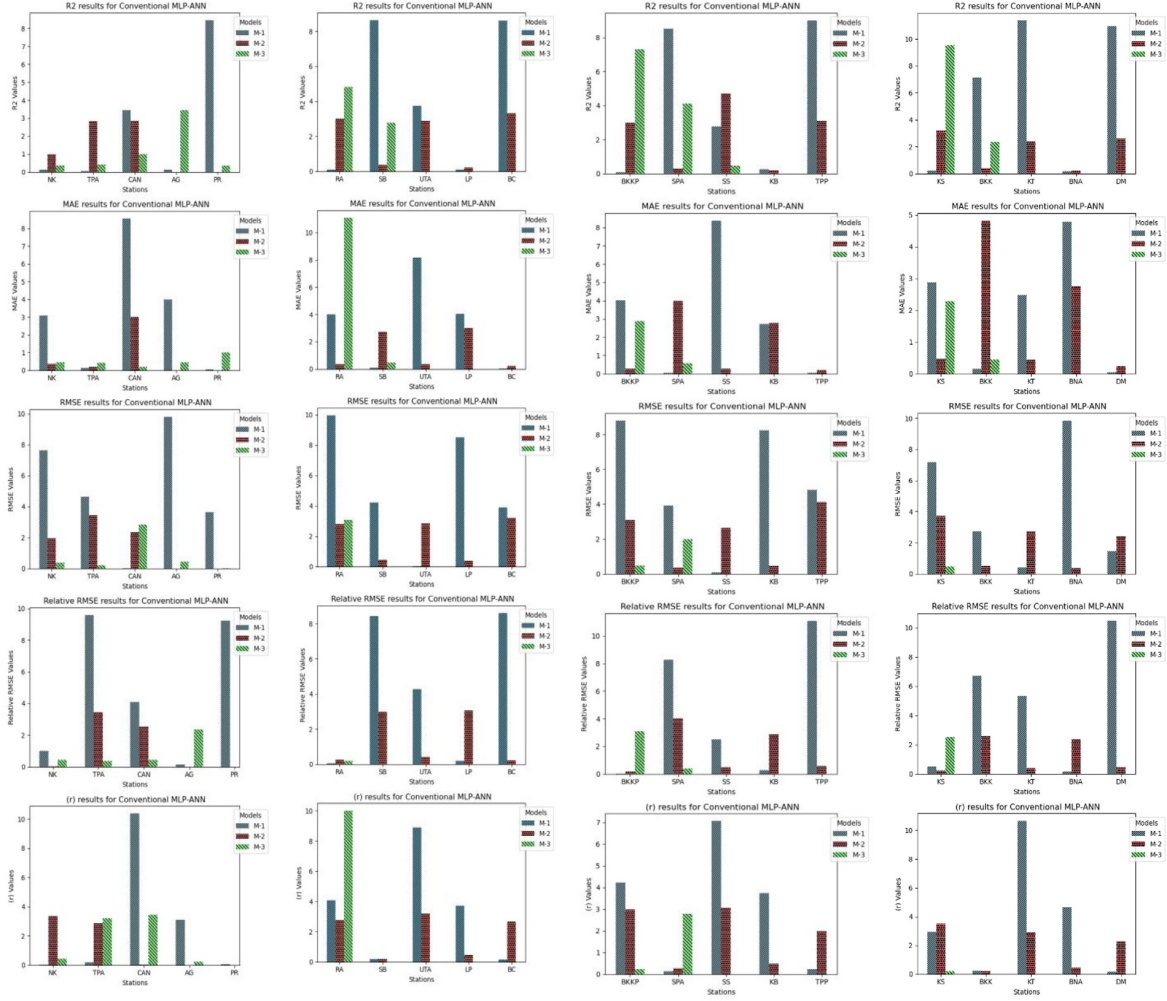
Comprehensive performance assessment of the developed prediction models (M-1, M-2, and M-3) utilizing Conventional MLP-ANN, conducted across TMD stations 1 to 20.

The assessment of three models (M-1, M-2, M-3), implemented through conventional deep MLP-ANN, as delineated in Fig. 12. Model M-2 stands out as a paragon of efficacy, notably demonstrated in forecasting precipitation for Station NK, where it achieves a remarkable R^2^ of 0.99. This near-perfect fit is complemented by a minimal MAE of 0.018 and RMSE of 0.046, underscoring its heightened accuracy. The RRMSE value of 1.96 further substantiates a reasonable relative error. In contrast, Model M-1 exhibits moderate performance with an R^2^ of 0.12, indicative of a comparatively weaker fit, while Model M-3 consistently positions itself between M-1 and M-2, suggesting a moderate level of performance. Across various stations, Model M-2 consistently outperforms Models M-1 and M-3 in critical metrics, including R^2^, MAE, and RMSE. Even in instances where Model M-1 occasionally exhibits commendable performance, especially when M-2 attains exceptionally high R^2^, the consistent superiority of M-2 is evident. Model M-3 consistently lags M-2 in accuracy metrics but maintains a performance commensurate with M-1. Examining specific stations, the trend persists. For example, at Station AG, Models M-1 and M-2 demonstrate comparable R^2^, yet M-2 excels in accuracy metrics, while Model M-3 lags. Similar patterns emerge at Stations PR and RA, where M-2 consistently surpasses M-1 and M-3 in R2, MAE, and RMSE. Even at Station SB, where M-1 attains the highest R^2^ (0.199), M-2 supersedes in terms of MAE and RMSE, indicating enhanced accuracy, while M-3 falls short in accuracy metrics. The consistent outperformance of Model M-2 is further emphasized across various stations, such as UTA, LP, BC, BKKP, SPA, SS, KB, TPP, KS, BKK, KT, BNA, and DM. M-2 consistently achieves higher R^2^ and lower MAE and RMSE metrics in each case than M-1 and M-3. It underscores the robustness and efficacy of M-2 in precipitation forecasting. While Models M-1 and M-3 occasionally demonstrate competitive performance, they consistently assume positions that are either inferior or intermediary to Model M-2. The conventional deep MLP-ANN architecture, as represented by Model M-2, is more adept at capturing the intricate relationships and temporal dependencies in precipitation data, leading to consistently superior forecasting accuracy. The superior performance of Model M-2 lies in its ability to effectively model the complex temporal patterns and relationships in precipitation data. The architecture of the conventional deep MLP-ANN, as represented by M-2, proves to be well-suited for the challenges posed by multivariate and lagged datasets, resulting in consistently superior forecasting accuracy across diverse observation stations and input combinations. Models M-1 and M-3, while showcasing occasional competitive performance, are consistently surpassed by the efficacy of Model M-2 in precipitation forecasting.

**Fig. 13:**
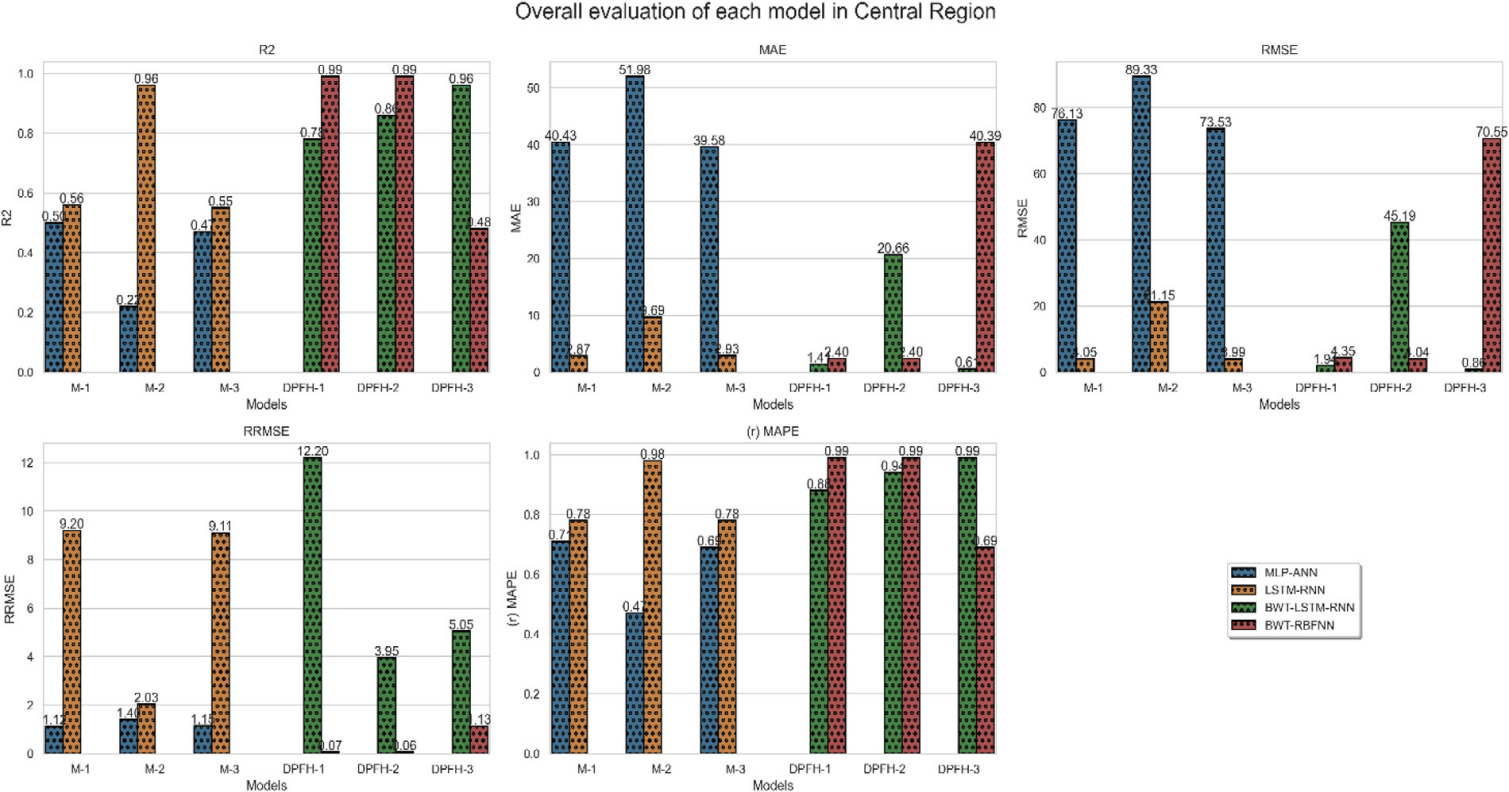
Comprehensive evaluation of forecasting models across the central region: aggregated performance metrics using the average observation dataset.

**Fig. 14:**
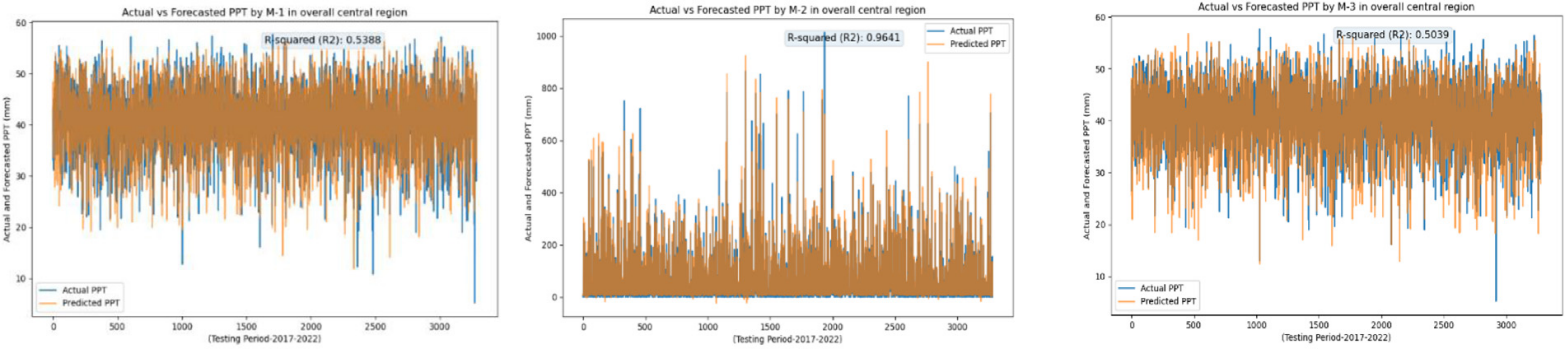
Actual vs forecasted precipitation during the testing period (2017-2022) by conventional LSTM-RNN with inputs combination-based models (DPFH-1, DPFH-2, and DPFH-3).

**Fig. 15:**
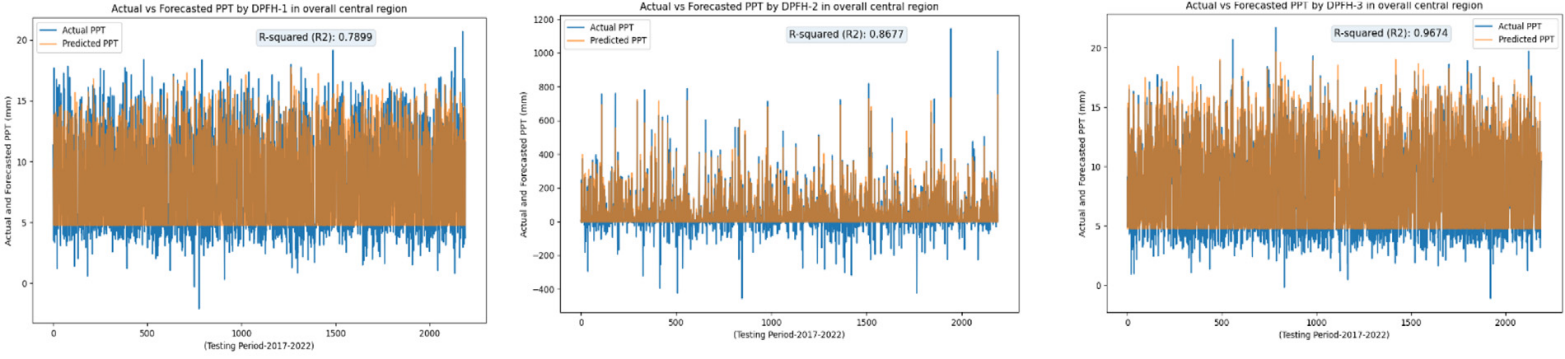
Actual vs forecasted precipitation during the testing period (2017-2022) by BWT-LSTM-RNN with inputs combination-based models (DPFH-1, DPFH-2, and DPFH-3).

**Fig. 16:**
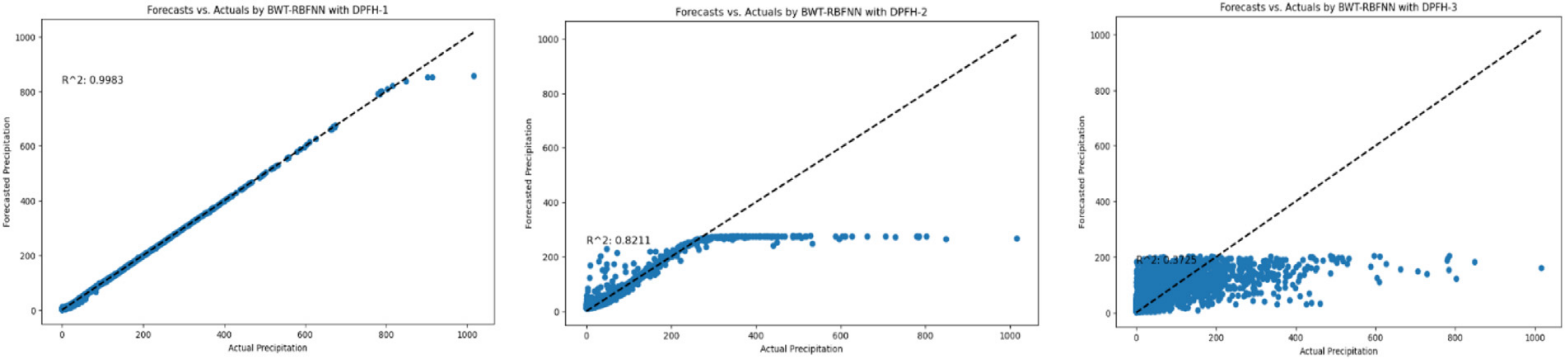
Actual vs forecasted precipitation during the testing period (2017-2022) by BWT-RBFNN with inputs combination-based models (DPFH-1, DPFH-2, and DPFH-3).

**Fig. 17:**
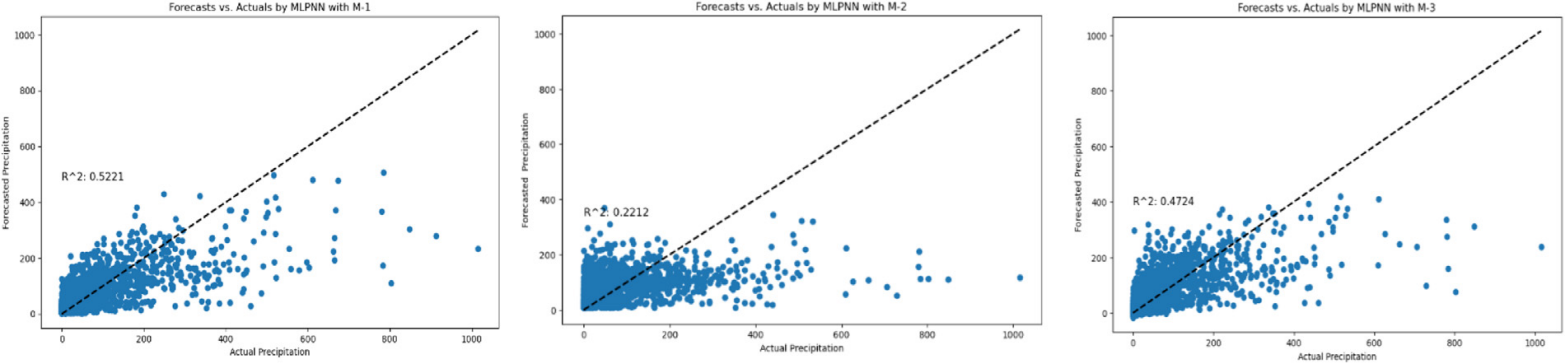
Actual vs forecasted precipitation during the testing period (2017-2022) by Conventional MLPNN with inputs combination-based models (M-1, M-2, and M-3).

Following an in-depth assessment of each model utilizing various input combinations at individual observation stations, the performance of hybrid and conventional precipitation prediction models was examined across the entire central region of Thailand. Fig. 13 presents the comprehensive evaluation of forecasting models across the central region. The MLP-ANN demonstrated moderate performance, with Model M-1 capturing half of the variance in precipitation and M-2 and M-3 displaying higher errors, indicating challenges in capturing complex patterns. In contrast, the LSTM-RNN exhibited notable performance improvements. Models M-1 and M-3 displayed reasonable R^2^, but M-2 stood out with a remarkable R^2^ of 0.96, showcasing LSTM-RNN's adeptness in handling univariate lagged datasets for precipitation forecasting. However, RRMSE values indicate room for enhancement, especially in M-2 and M-3. The integration of BWT-LSTM-RNN brought significant advancements. Across all three DPFH models, BWT-LSTM-RNN consistently outperformed other models, demonstrating superior R^2^ and substantially lower errors. Applying BWT with LSTM-RNN and considering multivariate lagged datasets collectively enhanced the model's adaptability, effectively capturing both short- and long-term dependencies. Conversely, the BWT-RBFNN models faced challenges, particularly in DPFH-3, where an R^2^ of 0.48 and elevated error metrics suggested limitations in capturing the intricate relationships in multivariate lagged datasets. The comparably higher RRMSE values further highlight the models' weaknesses. Additionally, Fig. 14, Fig. 15, Fig. 16, Fig. 17 depict the comparative analysis of actual versus forecasted precipitation across the central region throughout the testing period (2017-2022) for both conventional and hybrid models. The results exhibit the proficient performance of hybrid models across various input combinations, exhibiting alignment with the original dataset. The BDWT-LSTM-RNN notably demonstrated commendable accuracy, particularly evidenced by high R^2^ for all input configurations. The BWT-LSTM-RNN models emerged as the most robust and adaptive, excelling in capturing diverse precipitation patterns across different observation stations. Incorporating BWT and considering lagged datasets enhanced the model's accuracy and versatility, making it a superior choice for precipitation forecasting in hydrology.

### Discussion

Many studies have underscored that the performance of a standalone model, such as neural networks, can be increased by constructing hybrid models. Notably, splitting input and output data into sub-signals using the WT decomposition technique has been identified as superior to employing a single ANN [27,29,47,50,61,70]. The results from a comprehensive assessment of both standalone and hybrid models prove that the efficiency of hybrid models (LSTM-RNN and RBFNN) improved by utilizing the BWT methodology. This study's findings validate [71,72] that including input variables, namely Tmax, Tmin, RH, and WS, can increase prediction efficiency. It is crucial to emphasize that the influence of other variables on forecasted precipitation exhibits variations across different lead times and stations, as highlighted by [73].

The input-based model (M-1/DPFH-1), alongside their lagged datasets (M-3/DPFH-3) in BWT-LSTM-RNN models, has been empirically verified as advantageous for enhancing the accuracy predictions. Based on the comprehensive evaluation of all models using different input combinations (M-1/DPFH-1, M-2/DPFH-2, and M-3/DPFH-3) across 20 observation stations and in the combined central region, the BWT-LSTM-RNN model consistently demonstrated superior performance. Specifically, key evaluation metrics including R^2^, MAE, RMSE, RRMSE, and correlation coefficient (r) consistently surpassed threshold values in DPFH-3, incorporating a multivariate lagged dataset. DPFH-3 achieved remarkable results at station NK with an R^2^ of 0.98, a low MAE of 5.7, a high correlation coefficient (r) of 0.99, and an RMSE of 9.66. Similar trends were observed at stations such as TPA and UTA, where DPFH-3 consistently exhibited higher R^2^, MAE, and RMSE values than other models. Conversely, DPFH-1 and DPFH-2 displayed distinct performance patterns at each station despite utilizing different input combinations. DPFH-2 consistently demonstrated accurate and dependable precipitation forecasts characterized by low MAE and RMSE values, a near-perfect R^2^ close to 1.00, and consistent robust performance when utilizing a univariate lag dataset. In contrast, DPFH-1, leveraging multivariate input variables, exhibited mixed performance with a moderate R^2^ and higher MAE and RMSE values than DPFH-2 and DPFH-3. These results are strongly aligned with previous studies [27,29,47,50,61,70] which mentioned the superiority of hybrid models. The benefit of BWT is utilized by the BWT-LSTM-RNN model, improving the model's capacity to represent intricate temporal patterns in the precipitation data. The BWT-LSTM-RNN model demonstrates better capability in accurately depicting underlying dynamics within precipitation datasets by effectively capturing both short- and long-term trends and fluctuations. This capability addresses a common challenge faced by conventional LSTM-RNN models, which may struggle to accurately capture intricate temporal patterns, particularly when confronted with multivariate and lagged datasets (M-1 and M-3). However, the integration of WT within the BWT-LSTM-RNN model provides a solution to this limitation, enabling a more nuanced comprehension of temporal dynamics present in precipitation time series data. A critical analysis comparing the BWT-RBFNN with conventional models reveals architectural limitations. Despite its utilization of BWT, the BWT-RBFNN encounters challenges in managing the complexity arising from multivariate input combinations (DPFH-1 and DPFH-3). The incorporation of multiple variables and delayed datasets within these configurations may exacerbate issues of multicollinearity and redundant data, thereby impeding the model's ability to discern significant features essential for precipitation prediction. Moreover, the intricate linkages and temporal dependencies inherent in precipitation data present formidable obstacles for MLP-ANN models to comprehend effectively. Suboptimal performance can be attributed to the absence of dedicated mechanisms for handling sequential data and complex temporal patterns, particularly when compared to the advanced capabilities of the BWT-LSTM-RNN model. The BWT-LSTM-RNN model offers distinct advantages, particularly within the DPFH-3 configuration, by leveraging wavelet transforms to capture intricate temporal patterns and demonstrating adaptability to accommodate various input combinations. In contrast, LSTM-RNN, BWT-RBFNN, and MLP-ANN models confront limitations restricting their differing architectures, which not be optimally tailored to address the challenges inherent in multivariate and lagged datasets encountered in precipitation forecasting.

## Conclusion

This current investigation presents a hybrid method for daily precipitation forecasting for Thailand's central region, leveraging historical observations from 20 stations spanning the preceding 30 years. This approach incorporates three distinct models (M-1/DPFH-1, M-2/DPFH-2, and M-3/DPFH-3) and integrates univariate and multivariate meteorological factors as inputs to enhance the accuracy of precipitation prediction. To evaluate the effectiveness of the proposed method, thorough comparisons were conducted between it and the DPFH approach, along with traditional models such as the multilayer perceptron artificial neural network (MLP-ANN) and the long short-term memory recurrent neural network (LSTM-RNN). In the latter, the Biorthogonal Wavelet Transform (BWT) function was integrated with the radial basis function neural network (BWT-RBFNN) and LSTM-RNN (BWT-LSTM-RNN). The study encompassed multiple observation sites and comprehensively analyzed the performance of each model.

• The study found that LSTM-RNN outperformed MLP-ANN in precipitation prediction, particularly in detecting complex patterns within datasets with lagged variables. This indicates that LSTM-RNN has a better ability to capture temporal dependencies and intricate relationships within the data compared to MLP-ANN.
• Integration of BWT with LSTM-RNN resulted in significant advancements, consistently outperforming other models across different configurations. This integration enhanced the model's versatility, accuracy, and capability to capture both short- and long-term dependencies in the precipitation data. It suggests that the wavelet transform technique effectively complements LSTM-RNN in extracting relevant features from the data, leading to improved forecasting performance.
• The study identified limitations in both BWT-RBFNN and MLP-ANN architectures when dealing with multivariate and lagged datasets in precipitation prediction. Specifically, BWT-RBFNN encountered challenges in handling intricate interactions within multivariate lagged datasets, particularly evident in the DPFH-3 configuration. This suggests constraints in the model's ability to capture complex relationships in the data. Similarly, MLP-ANN exhibited moderate performance compared to LSTM-RNN, indicating its limitations in capturing the intricate temporal patterns present in the precipitation data.

Overall, the superior performance of the BWT-LSTM-RNN model can be attributed to its adept utilization of WTFs for feature extraction and its adaptability to multivariate input combinations. These findings underscore the importance of feature extraction techniques and model integration in improving the accuracy of precipitation forecasting. Additionally, the study highlights the potential of dynamic model adaptability and ensemble forecasting techniques for further enhancing forecasting performance, especially in regions with diverse climatic conditions.

### Future Directions

For future research, optimizing hybrid approaches, particularly those using the Biorthogonal Wavelet Transform (BWT), can potentially improve precipitation forecasting accuracy. Investigating the feasibility and efficacy of ensemble forecasting methodologies and dynamic model adaptation could provide valuable insights into improving forecasting models, particularly in regions with varying climatic circumstances. Also, it is suggested to incorporate large-scale climate variables as input for short-term precipitation forecasts. Such efforts would increase scholarly research in this sector and pave the way for more complex and effective precipitation forecasting systems.

## Limitations

The study's findings may not be readily applicable to regions outside Thailand due to potential variations in climatic conditions and topographical features. To employ this methodology for different regions, other wavelet transformation families must be applied to assess the more aligned family with the input data. Finally, it suggests extending the spatial-temporal analysis to capture localized precipitation variations by considering additional geographical and topographical features.

## Ethics statements

The data used in this research are secondary data derived from the office of the Thai Meteorological Department. It is a Deep learning computer-based method.

## Funding

No Funding was used in this Research.

## CRediT authorship contribution statement

**Muhammad Waqas:** Conceptualization, Methodology, Software, Formal analysis, Investigation, Writing – original draft. **Usa Wannasingha Humphries:** Formal analysis, Resources, Supervision, Writing – review & editing, Project administration, Funding acquisition. **Phyo Thandar Hlaing:** Formal analysis, Visualization. **Angkool Wangwongchai:** Data curation, Validation, Writing – review & editing. **Porntip Dechpichai:** Visualization, Supervision, Project administration.

## Declaration of competing interest

The authors declare that they have no known competing financial interests or personal relationships that could have appeared to influence the work reported in this paper.

## Data Availability

Data will be made available on request.
